# Estimates of population-level palliative care need in the UK: a descriptive analysis of mortality data before and during the COVID-19 pandemic

**DOI:** 10.1186/s12904-024-01574-5

**Published:** 2024-11-25

**Authors:** Erin Raquel Fantoni, Natasha Wynne, Anne M. Finucane

**Affiliations:** 1 Marie Curie, One Embassy Gardens, 8 Viaduct Gardens, London, SW11 7BW UK; 2 Clinical Psychology, School of Health in Social Science, University of Edinburgh, Edinburgh, EH8 9AG UK; 3 Marie Curie Hospice Edinburgh, 45 Frogston Road West, Edinburgh, EH10 7DR UK

**Keywords:** Mortality, Palliative care, Needs assessment, Health services needs and demand, Multimorbidity

## Abstract

**Background:**

Existing estimates of palliative care need in the UK were produced before the COVID-19 pandemic. We sought to produce updated, population-level estimates of palliative care need for each of the four UK nations and explore how these changed during the pandemic.

**Methods:**

We conducted a descriptive analysis of routine data. We used a well-established, diagnosis-based methodology which produced minimal estimates of palliative care need based on underlying causes of death; intermediate estimates based on underlying and contributory causes of death; and maximal estimates based on excluding unexpected causes of death. Additional estimates incorporated deaths involving COVID-19. All methods were applied to official mortality statistics from England, Wales, Scotland, and Northern Ireland for the years 2017 to 2021.

**Results:**

From 2017 to 2019 for the UK in total, palliative care need was estimated at ~ 74% (minimal), ~ 90% (intermediate) and ~ 96% (maximal) of total deaths, which was broadly consistent with previous studies. Results were similar across all nations. In the pandemic years, 2020-21, the minimal estimates remained stable in terms of the number of people in need but dropped significantly in terms of the proportion of deaths associated with palliative care need (to ~ 66%) due to the overall increase in mortality and large number of deaths from COVID-19. The intermediate (~ 90%) and maximal (~ 96%) estimates showed an increase in the number of people in need but remained stable in proportion of deaths. When deaths involving COVID-19 were treated as deaths associated with palliative need, the minimal estimates increased to ~ 77% and intermediate estimates increased to ~ 92%.

**Conclusions:**

In each of the UK’s nations, most people who die will have palliative care needs. Excluding deaths from COVID-19 in population-level estimates of palliative care need risks under-estimating true levels of need. Future studies which estimate population-level palliative care need should consider factoring in deaths from COVID-19.

## Background

Palliative care is a holistic, person-centred approach to improving the quality of life of patients with life-threatening, often terminal, illnesses and supporting their families [1]. It focuses on the prevention and relief of pain and other physical symptoms, as well as support for psychological or spiritual problems, and is delivered by a wide range of health and care professionals across varied settings [2, 3]. The provision of palliative care is an ongoing, strategic concern for service planners and policymakers across the UK. In England, the Health and Care Act (2022) places a legal requirement on Integrated Care Boards (ICBs) to commission palliative care services that meet their populations’ needs [4]. Wales has recently established a national End of Life Care Programme following the publication of its Quality Statement on palliative and end of life care [5, 6]. Work is underway to develop a new palliative care strategy in Scotland, while in Northern Ireland there have been calls for a new strategy to be approved as a matter of priority [7].

Estimating population-level palliative care need is essential to guide service planning and commissioning. Health and social care needs and needs assessment are conceptualised differently depending on the perspective adopted. Four categories of social need have been defined by Bradshaw [8]. ‘Normative’ need refers to professional or expert perceptions of need; ‘felt’ need refers to what individuals want; ‘expressed’ need is ‘felt’ need turned into action or demand, while ‘comparative’ need emphasises what is needed based on what others have or do not have and is used to assess needs both of individuals and areas. In order to inform palliative care strategies and commissioning plans, assessments of how many people need palliative care are typically based on normative need (a professional/expert perspective), with need being defined as the population’s ability to benefit from care [9].

A common estimate of need for high-income countries, such as the UK, is that 75% of people who die need palliative care [10, 11]. The main source for this is the observation by Gómez-Batiste et al. that more than 75% of the population in middle- and high-income nations will die from chronic, progressive conditions [10]. The same 75% estimate has been produced in England and Wales through a different methodology which used mortality and coroner service statistics to estimate how many deaths are unexpected and considered remaining deaths to have palliative care needs [12].

While 75% has been a useful estimate of chronic or predicted deaths overall, more detailed methodologies which consider both underlying (main) and contributory causes of death can be more sensitive to population shifts in mortality. The inclusion of contributory causes of death when estimating population palliative care need is especially important in countries such as the UK, where the ageing population has led to an increase in people dying from multiple health conditions [13]. Approaches based on both underlying and contributory causes of death, utilizing the International Classification of Disease – 10th Revision (ICD10) codes listed on death certificates, was initially developed by Rosenwax et al. [14] and then refined by Murtagh et al. [15] through consultations with medical professionals. These refined estimation methods have been widely applied in Australia, Europe, North America, and Asia [16–21]. They contain four approaches to estimating palliative care need using mortality and cause of death data [15]:

- First, a **minimal estimate** which considers *underlying* causes of death only.
- Second, a **lower intermediate estimate**, which includes all deaths in the minimal estimate, but also includes those with some *contributory* causes; and all individuals hospitalised in the year before death with the same condition as their recorded cause of death.
- Third, an **upper intermediate estimate**, which includes all deaths with any of the specific conditions in the minimal estimate recorded as either the underlying *or* a contributory cause on the death certificate.
- Fourth, a **maximal estimate** that includes all deaths except deaths from external causes (e.g.injury or poisoning) and those related to pregnancy, childbirth, puerperium period or postnatal period.

The first application of Murtagh’s method, which used English data from 2006 to 2008, produced a minimal estimate of palliative care need of 63% and intermediate estimates of 69% and 82% (see Table 1) [15]. As the mid-point between these two intermediate estimates, 75% was adopted in policy documents [22]. In more recent studies, the minimal estimate, which considers only the underlying cause of death, has risen to be broadly consistent with the often cited 75% estimate [11, 23, 24]. This is in part due to a significant rise in the number of deaths from dementia and Alzheimer’s disease, resulting from improved recording and diagnosis and updates to the framework used to code deaths [25]. The upper-intermediate estimate, which considers contributory causes of death, has risen to approximately 90% [23]. This estimate is increasingly important as multimorbidity becomes more prevalent in the UK, due to life expectancy increases and the ageing population [13, 26].

**Table 1:** Previous UK estimates of palliative care need using Murtagh et al. (2014) methodology

	Period	Population	Minimal	Intermediate (lower)	Intermediate (upper)	Maximal
**Murtagh et al. (2014)**	2006-08	England	63.0%	69.1%	81.9%	96.6%
**Marie Curie (2016)**	2012-14	England	74.3%	-	89.5%	95.9%
		Wales	73.6%	-	87.1%	95.9%
		Northern Ireland	73.6%	-	88.9%	94.7%
**Etkind et al. (2017)**	2011-2014	England and Wales	73.8%	-	-	-
			74.9%	-	-	-
**Finucane et al. (2021)**	2007-2017	Scotland	74.0%	-	88.0%	-
			74.0%	-	90.0%	-

The COVID-19 pandemic brought about significant changes in mortality patterns in the UK. The first wave of COVID-19 deaths in the UK began in March 2020 [27]. During its peak in April that year, the total number of deaths in England and Wales was more than twice normal levels [28]. A large proportion of these excess deaths had a registered cause of COVID-19, however deaths from other causes were also higher than the average from 2015 to 2019 [3]. A second wave in late 2020 again led to a higher number of deaths than the five-year average. Deaths from COVID-19 represented an even higher proportion of excess deaths in this wave, possibly reflecting more comprehensive testing at this stage of the pandemic. By May 2023, when the World Health Organisation (WHO) declared an end to the COVID-19 public health emergency, almost 227,000 people in the UK had died with COVID-19 listed as one of the causes on their death certificate [29].

In the UK, the pandemic had significant implications for the delivery of and demand for palliative and end of life care. A grey-paper review of dying, death and bereavement during COVID-19 emphasised the core role of palliative care in the pandemic response and recommended that palliative care should be available in all settings in which care is given to people with severe COVID-19 [3]. Palliative symptom control was deemed useful for people dying from COVID-19, though evidence on its effectiveness was lacking [30]. Internationally, palliative care triage plans and clinical guidance for patients dying with COVID-19 were developed [31, 32]. The National Institute for Clinical Excellence (NICE) published a rapid guideline for managing COVID-19 in March 2021, which included a section on palliative care [33]. NICE also published a clinical knowledge summary in 2022 which specifically addressed end of life care for people dying with symptoms or complications from COVID-19 infection [34]. Scottish Palliative Care Guidelines focused on symptom management for people dying from COVID-19 were also published and helped guide clinical practice during the pandemic [35].

Estimates of population-level palliative care need should be re-examined to take account of the significant changes in mortality patterns in recent years. Infectious and acute diseases such as COVID-19 are sometimes excluded from estimates of palliative care need. However, the consensus in clinical guidance is that palliative care is relevant for people dying from COVID-19 and this justifies exploration of population-level estimates of palliative care need with COVID-19 included.

The aims of this study are to:

1. Provide estimates of palliative care need in the four nations of the UK with the most recently available data (up to 2021).
2. Consider the impact of the pandemic on estimates of population-level palliative care need.
3. Compare estimates of population-level palliative care need when COVID-19 is included versus excluded as a cause of death associated with palliative care need.

In doing so, we provide an updated picture of the prevalence of palliative care need, accounting for mortality due to COVID-19, to inform policy and palliative care service development in the UK.

## Methods

### Design

We conducted a secondary analyses of routinely available data on death registrations from England, Northern Ireland, Scotland and Wales to estimate national palliative care need. We produced three levels of estimates for 2017–2021 for each nation using the methodology for the minimal, upper intermediate and maximal estimates from Murtagh et al. [15] This methodology was chosen because it is well established and the requisite data can be readily accessed. The lower intermediate method included in Murtagh et al. was not used as the data required to generate this estimate is not routinely available. For simplicity, as there is only one intermediate estimate in our paper, it is referred to as the ‘intermediate estimate’ rather than the ‘upper intermediate’ estimate. We calculated:

1. A minimal estimate which considered underlying causes of death only.
2. An intermediate estimate which included all deaths with any of the specific conditions in the minimal estimate recorded as either the underlying *or* a contributory cause on the death certificate.
3. A maximal estimate which excluded deaths from external causes and those related to pregnancy, childbirth, the puerperium period or the postnatal period.

For full details of the ICD-10 codes and for each of these estimates, see Table 2.

We first produced these figures using the ICD-10 diagnostic codes previously used to estimate palliative need [11, 15] (see Table 2). Then, we created additional estimates with COVID-19 codes (U07.1 and U07.2) included as a cause of death associated with palliative care need.

**Table 2:** ICD-10 codes and diagnoses for estimating Palliative Care need [11, 15]

Estimate	Method	
**Minimal**	All deaths with any of the following recorded as the underlying cause on the death certificate:	
		**ICD-10 Codes**
	Malignant neoplasm	C00-C97
	Heart disease and heart failure	I00-I52 (excl. I12 & I13)
	Reno-vascular disease, renal failure	I12, I13, N17, N18, N28
	Haemorrhagic, ischaemic and unspecified stroke	I60-I69
	Liver disease	K70-K77
	Chronic lower respiratory disease, respiratory failure	J40-47, J96
	Huntingdon’s disease, motor neurone disease, Parkinson’s disease, progressive supranuclear palsy, multiple sclerosis, multi system atrophy	G10, G12.2, G20, G23.1, G35, G90.3
	Dementia, vascular dementia, Alzheimer’s disease, senility	F01, F03, G30, R54
	HIV	B20-24
	COVID-19*	*U07.1*,* U07.2*
Lower intermediate*	All deaths in the minimal estimate, but also includes those in which Alzheimer’s, dementia, senility, or chronic renal failure are listed as a contributory cause; and all individuals hospitalised in the year before death with the same condition as their recorded cause of death.	
Upper intermediate**	All deaths with any of the specific conditions in the minimal estimate recorded as either the underlying *or* a contributory cause on the death certificate.	
**Maximal**	All deaths except those from any of the following underlying causes:	
	Injury, poisonings, and certain consequences of external causes	S00-T98
	External causes of morbidity and mortality (accidents, self-harm, assault, etc.)	V01-Y98
	Conditions from pregnancy, childbirth, or puerperium period	O00-O99
	Certain conditions originating during the perinatal period	P00-P96

*Note: The lower intermediate estimate is not used in this study. This study used only the “upper intermediate estimate” and for simplicity it is referred to as the “intermediate estimate" throughout the paper

**Note: COVID-19 codes are only included in alternative estimates in the present study where clearly labelled as including codes for COVID-19 deaths

### Data

#### Death registrations

English and Welsh data for annual death registrations for 2017–2021 were provided by the Office for National Statistics (ONS). For Northern Ireland, the data were provided by the Northern Ireland Statistics and Research Agency (NISRA). For Scotland, the data were provided by the National Records of Scotland (NRS).

#### Estimates using methodology from Murtagh et al. [15]

1. For the minimal estimate, data were publicly available for England, Scotland and Wales. A data request had to be placed with NISRA for Northern Ireland due to the three-digit ICD-10 codes used in the estimates not being routinely published.
2. For the intermediate estimate, bespoke requests were placed with ONS, NISRA and the NRS.
3. For the maximal estimate, data were publicly available for all four nations.

#### Estimates with the ICD-10 codes for COVID-19 included

COVID-19 is recorded on death certificates using one of two allocated emergency codes: U07.1 is used when COVID-19 has been confirmed by laboratory testing; U07.2 is used when COVID-19 has been diagnosed clinically or epidemiologically, without a conclusive laboratory test [36]. To produce estimates that included COVID-19 deaths, the codes U07.1 and U07.2 were both incorporated into the list of codes shown in Table 2 to create COVID-inclusive minimal and intermediate estimates. The maximal estimates were not reproduced as, through their method of exclusion, they already contained COVID-19 deaths.

1. For the minimal estimate, data including the ICD-10 codes for COVID-19 were publicly available for all four nations.
2. For the intermediate estimate bespoke requests were placed with ONS, NISRA and the NRS.

### Analysis

#### Estimates of current need

For the minimal, intermediate and maximal estimates, the number of deaths with relevant ICD-10 codes were compiled into tables in Microsoft Excel for ease of analysis, and proportions were computed based on comparing the total deaths with relevant ICD-10 codes to the total number of deaths. Tables for the conventional estimates and tables for the estimates which incorporated COVID-19 death codes were produced separately.

## Results

### Estimates where COVID-19 is not included as a cause of death associated with palliative care needs

First the conventional methodology (meaning COVID-19 death codes were not included in the list of codes considered to need palliative care) was used. Across nations, the minimal, intermediate and maximal estimates were largely consistent. Across time, there was a clear impact of the pandemic, so estimates are described separately in-text for pre-pandemic (2017–2019) and pandemic (2020–2021) years.

Minimal estimates of palliative care need from 2017 to 19 across all four nations ranged from 72.8% (Northern Ireland, 2018) to 74.6% (England, 2017). For the intermediate estimates, the range was 87.7% (Wales, 2017) to 90.7% (Northern Ireland, 2019) and for the maximal estimates, it was 93.5% (Scotland, 2019) to 96.1% (England, 2020) (see Table 3).

For 2020-21, which was impacted by pandemic-related deaths, deaths needing palliative care based on minimal estimates were similar in raw numbers to previous years but were proportionally smaller. The range for the minimal estimates was 65.6–67.6%. The intermediate and maximal estimates increased in raw numbers in 2020-21, but proportionally remained steady compared to previous years. The range for intermediate estimates was 88.1–90.6% and the range for the maximal estimates was 94.1–96.7%. For exact figures for all conventional estimates across time and nations, see Table 3.

**Table 3:** Conventional estimates of deaths with palliative care needs: 2017–2021

		Minimal		Intermediate		Maximal		Total Deaths	
Country	Year	Frequency	%	Frequency	%	Frequency	%		
**England**									
	2017	372,056	74.58%	451,755	90.55%	479,395	96.09%		498,882
	2018	373,919	73.92%	457,898	90.52%	484,470	95.77%		505,859
	2019	368,536	74.24%	449,910	90.64%	475,267	95.75%		496,370
	2020	375,313	66.11%	513,569	90.46%	549,015	96.71%		569,700
	2021	362,444	65.98%	495,307	90.16%	527,828	96.08%		549,349
**Northern Ireland**									
	2017	11,701	72.97%	14,520	90.55%	15,048	93.84%		16,036
	2018	11,595	72.82%	14,378	90.30%	14,911	93.65%		15,922
	2019	11,551	73.30%	14,289	90.68%	14,770	93.73%		15,758
	2020	11,831	67.17%	15,960	90.61%	16,651	94.53%		17,614
	2021	11,520	65.61%	15,850	90.27%	16,588	94.48%		17,558
**Scotland**									
	2017	42,822	73.98%	51,965	89.78%	54,629	94.38%		57,883
	2018	42,653	72.91%	51,978	88.85%	55,011	94.03%		58,503
	2019	42,493	73.13%	51,691	88.96%	54,344	93.52%		58,108
	2020	42,478	66.28%	56,849	88.70%	60,293	94.07%		64,093
	2021	42,982	67.60%	56,211	88.40%	59,820	94.08%		63,587
**Wales**									
	2017	24,452	73.54%	29,159	87.70%	31,887	95.91%		33,248
	2018	25,118	73.00%	30,208	87.80%	32,994	95.90%		34,406
	2019	24,432	73.63%	29,267	88.20%	31,845	95.97%		33,183
	2020	24,823	66.37%	32,965	88.14%	36,179	96.74%		37,399
	2021	24,299	67.25%	32,093	88.81%	34,724	96.10%		36,135
**UK**									
	2017	451,031	74.42%	547,399	90.32%	580,959	95.86%		606,049
	2018	453,285	73.74%	554,462	90.20%	587,386	95.56%		614,690
	2019	447,012	74.08%	545,157	90.34%	576,226	95.49%		603,419
	2020	454,445	65.98%	619,343	89.92%	662,138	96.13%		688,806
	2021	441,245	66.19%	599,461	89.92%	638,963	95.85%		666,629

*Note: Minimal estimate considers only underlying cause of death. The intermediate includes the underlying and contributory causes of death. The maximal excludes underlying causes of death deemed to be unexpected*

### Estimates with COVID-19 included as a cause of death associated with palliative care needs

Estimates were reproduced with the ICD-10 codes for COVID-19 included as deaths requiring palliative care. As a result, the minimal estimates increased notably (range of increase from 6.4 to 11.9%) to become 72.1-78.1%. Intermediate estimates increased by a smaller amount (range of increase from 1.5 to 3.0%) to become 90.2-92.6%. Maximal estimates were not reproduced as the conventional methodology already included COVID-19 deaths due to its approach of estimating through exclusion of some codes. For exact figures for all estimates that include COVID-19 death codes in the methodology in 2020 and 2021 for all nations, and the percentage point increase in each estimate, see Table 4.

**Table 4:** Impact of including COVID-19 in Palliative Care need estimates

		Minimal Estimates											Intermediate Estimates					
		Without COVID-19 Codes						With COVID-19 Codes					Without COVID-19 Codes			With COVID-19 Codes		
Country	Year	Total Deaths		Frequency with palliative needs		%		Frequency with palliative needs		%	Increase*		Frequency with palliative needs		%	Frequency with palliative needs	%	Increase*
England	2020	569,700		375,313		**66.11%**		444,612		**78.04%**	11.93%		513,569		**90.46%**	526,896	**92.49%**	2.03%
	2021	549,349		362,444		**65.98%**		425,998		**77.55%**	11.57%		495,307		**90.16%**	508,802	**92.62%**	2.46%
Northern Ireland	2020	17,614		11,831		**67.17%**		13,456		**76.39%**	9.22%		15,960		**90.61%**	16,215	**92.06%**	1.45%
	2021	17,558		11,520		**65.61%**		12,650		**72.05%**	6.44%		15,850		**90.27%**	16,176	**92.13%**	1.86%
Scotland	2020	64,093		42,478		**66.28%**		48,526		**75.71%**	9.43%		56,849		**88.70%**	57,884	**90.31%**	1.61%
	2021	63,587		42,982		**67.60%**		47,815		**75.20%**	7.60%		56,211		**88.40%**	57,334	**90.17%**	1.77%
Wales	2020	37,399		24,823		**66.37%**		29,205		**78.09%**	11.72%		32,965		**88.14%**	34,082	**91.13%**	2.99%
	2021	36,135		24,299		**67.25%**		27,949		**77.35%**	10.10%		32,093		**88.81%**	33,046	**91.45%**	2.64%
								Average increase:			9.75%					Average Increase:		2.10%
UK	2020	688,806		454,445		**65.98%**		535,799		**77.79%**	11.81%		619,343		**89.92%**	635,077	**92.20%**	2.28%
	2021	666,629		441,245		**66.19%**		514,412		**77.17%**	10.98%		599,461		**89.92%**	615,358	**92.31%**	2.39%

## Discussion

We provide estimates of palliative care need across the four nations of the UK, consider the impact of the COVID-19 pandemic on these estimates, and demonstrate the impact of adding COVID-19 ICD-10 codes to produce the estimates.

### Population level estimates of palliative care need when deaths from COVID-19 are excluded

The minimal estimate results from before the pandemic (2017–2019) broadly aligned with the oft-used 75% estimates. (15, 22). However, after the onset of the pandemic (2020–2021), the minimal estimate falls below 75% (unless deaths from COVID-19 are also counted as needing palliative care). During the initial waves of the pandemic, COVID-19 caused an increase in deaths, and many of the people who died with COVID-19 also had pre-existing, chronic conditions considered to benefit from palliative care, such as dementia, heart disease and chronic lower respiratory diseases [37, 38]. The minimal estimates are not able to demonstrate this increase in deaths amongst people with palliative care need as they do not account for any co-morbid conditions among those who died of COVID-19. This means that the minimal estimates likely underestimate palliative care need.

In contrast, the increase in deaths during the pandemic was reflected in the intermediate estimates, with these estimates showing more people in need of palliative care during the pandemic years than pre-pandemic. Even though COVID-19 was not included as a cause of death requiring palliative care in these estimates, the increase in need was due to a high proportion of the people who died with COVID-19 also having chronic health conditions that would benefit from palliative care [37–39]. The intermediate estimates’ ability to account for underlying or contributory causes of death makes them more robust estimates of need than the minimal estimates.

### The inclusion of COVID-19 ICD-10 codes to estimate palliative care need

We produced alternative estimates by adding ICD-10 codes for deaths due to COVID-19 to the codes typically used in the literature [11, 15]. In these, the minimal estimates increased substantially (~ 10%), and the intermediate estimates increased slightly (~ 2%). The differing impacts on the minimal and intermediate estimates are because the conventional intermediate estimates, which account for contributory causes of death, already capture many COVID-19 deaths due to the high rates of co-morbidities in people who die with COVID-19 [39].

As in previous epidemics and pandemics, hospice and palliative care services played an essential role in the response to COVID-19 [40]. Research literature developed around the provision of palliative care for patients with COVID-19, with some advocating for palliative care consultations as standard procedure for patients hospitalised due to the illness [41–43]. In May 2023, the WHO declared that COVID-19 had become an established and ongoing health issue and no longer constituted a public health emergency [44]. While COVID-19 was the leading cause of death in England and Wales in 2021, it had fallen to the sixth leading cause in 2022, and more deaths were routinely reported from influenza than COVID-19 by 2023 [45]. Some may be concerned that including COVID-19 ICD-10 codes in estimates of population palliative care need will produce an overestimate, and that COVID-19 codes should be excluded as other acute diseases are sometimes excluded. However, there is no clear consensus on inclusion or exclusion of some acute diseases. For instance, the first iterations of the Murtagh methodology for estimating palliative care need included ICD-10 codes for influenza and pneumonia (J09-J18) [15]. However, others excluded these acute diseases [11, 19]. More recently, a study estimating palliative care needs in Japan has included codes relating to influenza, pneumonia and upper and lower respiratory tract infections, though the paper’s discussion acknowledges lingering uncertainty about their inclusion [21]. Despite the lack of consensus on which codes to include, given that palliative care interventions are recommended for symptom control for people dying from COVID-19 [33, 34] we suggest that COVID codes should be included in future estimates of palliative care need.

### The importance of accounting for multimorbidity

We believe that using an estimate of palliative care need which accounts for both underlying and contributory causes of death, including COVID-19, is optimal. The UK’s population is aging, which has led to an increase in chronic health conditions with more people dying as a result of more than one condition [13, 23]. The minimal estimates are unable to account for this growing demographic as they focus only on underlying cause with no consideration of contributory causes. For example, a person who dies as a result of a fall, with COVID-19 as a contributory cause, would not be identified as having a palliative care need, though we argue that such a need may exist. Thus, intermediate estimates are more accurate as they take account of contributory causes as well as underlying causes. Given their comprehensiveness, intermediate estimates may be more useful for planning palliative and wider health and care services which are oriented towards treating individuals with complex, co-existing needs, including COVID-19, rather than delivering disease-based models of care [46]. Such an orientation is important, as there is research to support that patients with multiple, life-limiting illnesses receive less care towards the end of life than is merited by the overall burden of their illnesses [47].

### Limitations

The estimates in this paper are based on publicly available, official mortality statistics that are robust and straightforward to analyse. However, there are limitations.

First, the use of cause of death data as indicators of palliative care need relies on death certificates being completed accurately. There is evidence that during the disruption of the pandemic, there was a slight fall in the quality of certification for non-COVID-19 deaths, with more deaths recorded from ill-defined underlying causes such as “old age” [48]. This may contribute to an under-estimate of palliative care need, particularly in the minimal estimates as they only consider underlying causes of death.

Second, the use of annual mortality statistics means that people who would benefit from palliative care more than a year before their death are not included, further underestimating need. These estimates therefore only tell us how many people die with palliative care needs each year, not how many people are living with palliative care needs. Though palliative care is often delayed until the final weeks or days of life [49], there is a strong case for identifying need and integrating palliative care earlier, potentially at any stage after the diagnosis of a life-limiting condition [50].

Finally, while providing population-level estimates can inform service development, it does not provide insight into palliative care needs at an individual level. To do this, individual level assessment of patients is required. Further, our analysis cannot by itself show the extent of palliative care needed in terms of resource or specific service implications. Research to determine the complexity of individual-level palliative care needs and inform more systematic care planning is ongoing [51, 52].

### Future research

Expert consultation and consensus building about if and how deaths from COVID-19 and similar conditions should be included in mortality-based estimates of population-level palliative care need would be valuable and allow a consensus to emerge that would support international comparisons. Specifically, a consensus on which disease codes are linked with palliative care need would be useful.

While the estimates here provide a count of individuals, the estimates are not able to quantify how much care is needed by each person or for people dying as a result of different diseases. Future research that details what level of care is typically needed for the included diagnoses, especially in the context of multi-morbidity, would provide policy makers and commissioners with a more precise understanding of the service and financial implications of meeting need. Similarly, future analysis might also describe in more detail the conditions people with palliative care needs died from, as well as their characteristics such as age and gender.

There is a lack of robust data to provide an overall estimate of how many people need palliative care but receive no or inadequate palliative care. Barriers to accessing palliative care have been identified in some population groups, including those aged over 85 years, those with non-malignant conditions, and minority ethnic communities [53], yet population-level data is lacking. Research to develop methods to estimate unmet palliative care need would be useful and would help prioritise areas for service development and commissioning. There have been some efforts to use surveys of providers [54] and General Practice palliative care registers [55] to estimate how many people access palliative care services, but neither can indicate whether services effectively meet people’s needs once accessed. A clearer understanding of how much current need is unmet would provide impetus for expanding palliative care in the UK. Further identifying in which populations need most often goes unmet would also allow for any expansions of care to be carefully targeted and thus as effective as possible.

## Conclusion

Across every nation of the UK, most people who die each year would benefit from palliative care. The pandemic had a profound effect on health and healthcare services, with a significant increase in mortality in 2020 and 2021. Methodologies which use mortality data to estimate population palliative care need typically exclude infectious diseases such as COVID-19; however, this study suggests that excluding deaths from COVID-19 from estimates of palliative care need risks under-estimating true levels of need in the population. This is a particular risk for estimate methodologies which only consider underlying causes of death. Researchers should therefore explicitly consider how they will factor in deaths from COVID-19 when producing future estimates of population-level palliative care need.

## Data Availability

Data for England’s and Wales’ minimal and maximal estimates are available on the ONS webpage here, through viewing files “2021 edition of this dataset” through “2017 edition of this dataset”: Deaths registered in England and Wales - Office for National Statistics (ons.gov.uk). Data for England’s and Wales’ intermediate estimates are available on the ONS webpage here- De-duplicated counts of deaths by selected causes, 2017 to 2021 - Office for National Statistics (ons.gov.uk). Data for Scotland’s minimal and maximal estimates for 2017–2019 are available on the NRS webpage for vital events through viewing files “vital events reference Table 2017” to “vital events reference Table 2019” Archive - Historical data back to 2001 | National Records of Scotland (nrscotland.gov.uk). Data for 2020 is available here: Vital Events Reference Table 2020 | National Records of Scotland (nrscotland.gov.uk). Data for 2021 is available here: Vital Events Reference Table 2021 | National Records of Scotland (nrscotland.gov.uk). Data for Northern Ireland’s maximal estimates are available on the NISRA webpage: Death Statistics | Northern Ireland Statistics and Research Agency (nisra.gov.uk). Data for the Scotland intermediate estimate and the Northern Ireland minimal and intermediate estimates were retrieved through bespoke data requests to NRS and NISRA, respectively, that were not subsequently published on the providers’ websites. They are available from the corresponding author on reasonable request.

## Abbreviations

NISRA: Northern Ireland Statistics and Research Agency
NRS: National Records of Scotland
ONS: Office for National Statistics
